# Prediction of Giant Thermoelectric Power Factor in Type-VIII Clathrate Si_46_

**DOI:** 10.1038/srep07028

**Published:** 2014-11-13

**Authors:** Payam Norouzzadeh, Charles W. Myles, Daryoosh Vashaee

**Affiliations:** 1 Helmerich Advanced Technology Research Center, Oklahoma State University, Tulsa, OK 74106, USA; 2 Department of Physics, Texas Tech University, Lubbock, Texas 79409-1051, USA; 3 Department of Electrical and Computer Engineering, North Carolina State University, Raleigh, NC 27606, USA

## Abstract

Clathrate materials have been the subject of intense interest and research for thermoelectric application. Nevertheless, from the very large number of conceivable clathrate structures, only a small fraction of them have been examined. Since the thermal conductivity of clathrates is inherently small due to their large unit cell size and open-framework structure, the current research on clathrates is focused on finding the ones with large thermoelectric power factor. Here we predict an extraordinarily large power factor for type-VIII clathrate Si_46_. We show the existence of a large density of closely packed elongated ellipsoidal carrier pockets near the band edges of this so far hypothetical material structure, which is higher than that of the best thermoelectric materials known today. The high crystallographic symmetry near the energy band edges for Si_46_-VIII clathrates is responsible for the formation of such a large number of carrier pockets.

The good thermoelectric materials that have been studied so far have shown several features proposing some material design rules. Some have shown lattices with high average coordination number, a high dielectric constant, a low average electronegativity difference between the atoms, and a band gap more than ~10k_B_T where T is the operating temperature of the thermoelectric material^1^. Some have a large unit cell and contain both atoms of heavy elements and atoms with large spin-orbit coupling^2^,^3^,^4^. It is also desirable to have materials composed of more than one element (such as binaries, ternaries, etc.), high crystal symmetry (only materials with high crystal symmetry possibly have degenerate peaks or valleys in their band structure), materials with electronic structure producing a rapidly changing density of states close to the band edges, and a multivalley band structure with valleys away from the Brillouin zone boundaries^5^,^6^,^7^,^8^,^9^,^14^,^21^. The multiplicity (degeneracy) of the band extrema is the number of equivalent peaks in the valence band or valleys in the conduction band. In general, any carrier pocket that is close to the Fermi energy (within a few k_B_T) contributes to the TE power factor; consequently, the presence of a large number of such valleys can result in an enhancement of ZT^10^. If the valence or the conduction band extrema are located outside of the center of the Brillouin zone, a multi-valley band structure forms, which is the case for many of the best known thermoelectric materials. In practice, it is only for optimum doping concentrations, where the Fermi energy is close to the band extrema, that a multi-valley band structure has a serious effect on the thermoelectric efficiency.

The sheer number of material structures that have been studied in the search for good thermoelectric properties is vast. Most of the new promising thermoelectric (TE) materials can be categorized into two general groups. These are materials with small thermal conductivity or the materials with a high TE power factor (i.e. Seebeck coefficient squared times electrical conductivity). The first group of materials includes complex crystal structures that yield low lattice thermal conductivity such as FeCo_3_Sb_12_^11^, Yb_14_MnSb_11_^12^, Ba_8_Ga_16_Ge_30_^13^, Zn_4_Sb_3_^14^, or Ag_9_TlTe_5_^15^, or nanostructured materials that reduce the thermal conductivity more than the electrical conductivity^16^,^17^,^18^,^19^,^20^,^21^. Examples of the second type of materials include engineered energy band structures with increased number of energy band minima close to the Fermi surface, such as PbTe_0.85_Se_0.15_:Na^22^ or with sharp features in the density of states close to the band edge such as Tl_0.02_Pb_0.98_Te^23^ that have resulted in the enhancement of the TE power factor^24^. For some other cases such as ErAs: InGaAs/InGaAlAs superlattices, the enhancement in ZT is due to the increase of the Seebeck coefficient due to the hot carrier energy filtering^25^,^26^.

While the first direction has resulted in a larger number of good thermoelectric materials^10^,^21^,^27^,^28^,^29^, when the thermal conductivity of the lattice becomes comparable to (or smaller than) that of the charge carriers, the enhancement of the figure-of-merit (ZT) becomes less significant. Hence, to further improve ZT, an increase in the power factor simultaneously with a reduction of the thermal conductivity is necessary.

To this end there is no clear roadmap for finding materials that can satisfy both needs and theoretical studies from first principles can significantly help in this materials search. In practice, the synthesis and characterization of many samples are required to find the optimal processing parameters that may or may not result in a high value of ZT. Therefore, theoretical predictions can save significant time and effort prior to the experimental investigation of such material structures.

Among the complex materials that can have low thermal conductivity are clathrate materials^30^. In these material systems, besides the fact that the open framework of clathrates reduces the lattice thermal conductivity considerably^31^, the guest atoms are considered as another source of thermal conductivity reduction^30^. The guest atoms are relatively free to rattle in the framework (or the cage) due to their weak interaction with the host. This localized rattling of the guest atoms is believed to resonantly scatter the acoustic phonon modes from the framework, which results in a reduction of the lattice thermal conductivity^32^.

We have recently found that the pristine Si_46_ type-VIII clathrate material possesses a large density of carrier pockets near the band edges that would increase the electronic density of states (DOS) in that region^33^. In general, the contribution of multiple conduction (or valence) bands to form a high degeneracy of carrier pockets near the Fermi energy, without a significant reduction in the carrier mobility, is considered as an effective method to enhance the thermoelectric power factor^22^.

Considering that the clathrate structures have shown small thermal conductivity, one would expect Si_46_ type-VIII clathrate has a large ZT^34^,^35^. Nevertheless, other techniques such as intercalation by guest atoms^36^,^37^,^38^, nanostructuring, or the combination of both methods can be pursued to reduce the thermal conductivity of the pristine Si_46_ type-VIII. Since the guest atoms are loosely bonded to the framework, their effect on the electronic properties of the host material is small. The nanostructuring method to produce materials with enhanced ZT values is specially gaining popularity and is widely applicable to the bulk materials^39^ although some studies have shown that the bulk nanostructuring may have a negative effect on the figure-of-merit ZT in some materials^40^. Therefore, combining the concepts of bulk nanostructuring and enhanced DOS near the band edges in Si_46_-VIII may result in both a low thermal conductivity and a large power factor.

In the present study, we report the results of an investigation of the charge transport properties of crystalline type-VIII clathrate Si_46_. We have studied the role of each band extremum close to the Fermi energy. We have used a multi-band semi-empirical approach which considers the characteristics of different carrier pockets. The thermoelectric properties have been calculated for both n-type and p-type materials as functions of both doping concentration and temperature. The results showed that the predicted extra-large number of carrier pockets of both n and p type structures can potentially lead to very large power factor^41^,^42^.

The details of the multiband Boltzmann transport code are presented elsewhere^20^. The main input parameters needed for this model are the band structure parameters, doping concentrations and the temperature. The physical parameters are listed in Table 1 of the supplementary information. The material band structure and other transport parameters were calculated from first principles in ref. 33. We did not calculate the thermal conductivity, however, it is good to know that the empirical value of thermal conductivity for type-II Si_136_ clathrate is 2.5 W/mK at room temperature^35^. Such a low thermal conductivity is expected for other elemental Si clathrates which in turn can potentially result in high performance thermoelectric compounds. We applied an integrated first principles-semi classical model to calculate electrical transport properties of the type-VIII clathrate Si_46_ and to obtain quantitatively reliable predictions.

## Results

**Figure 1:**
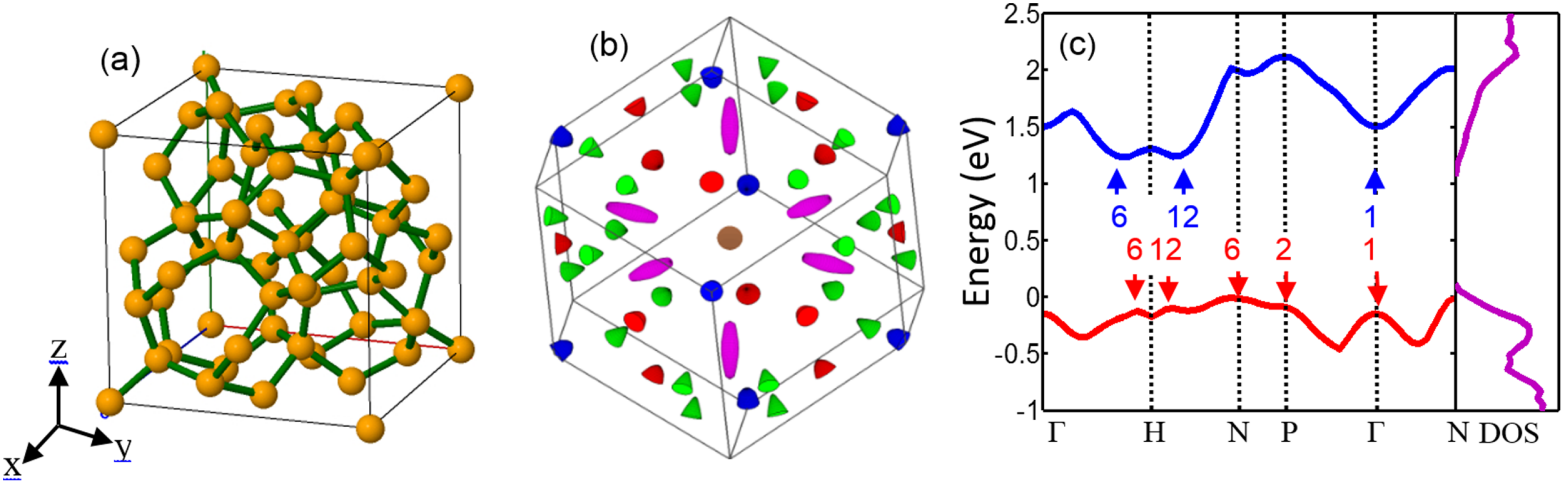
(a) Crystal structure of the type-VIII clathrate Si_46_ in real space. (b) Brillouin zone of the Si_46_ type- VIII clathrate showing the hole pockets at Γ = (0, 0, 0) point (brown), on the ΓH line (violet), on the NH line (green), at P = (1/4, 1/4, 1/4) point (blue), and at N = (1/2, 0, 0) points (red). The valley degeneracies for Γ, N, P, ΓH and NH are 1, 6, 2, 6, and 12, respectively. (c) The predicted conduction and valance band structures and the densities of states. The numbers with arrows indicate the multiplicity of each extrema. The ellipsoidal curvatures do not present the actual effective masses. The mass values are presented in the supplementary file.

Figure 1(a–c) presents the crystal lattice structure, the Brillouin zone with an interestingly large density of carrier pockets near the valance band edge, the electronic band structure and the density of states of this clathrate. The band structure is complex with multiple extrema in both the valence and conduction bands. Hence, the DOS of Si_46_-VIII varies with a larger slope near the band edges, for example, compared with that of the diamond structured Si. Figure 1-(b) shows the Brillouin zone of this material with degenerate hole pockets at the Γ, N, and P points and along the ΓH and NH lines. The figure presents 6 pockets along the ΓH line which are completely inside the Brillouin zone and 24 half-pockets along the NH line (green). Moreover, it predicts one pocket at the Γ = (0, 0, 0) point, 8 quarter-pockets at P = (1/4, 1/4, 1/4) points and 12 half-pockets at N = (1/2, 0, 0) points. Therefore, the degeneracies of the Γ, N, and P points and along the ΓH and NH lines are 1, 6, 2, 6, and 12, respectively, which add up to 27. This is significantly higher than that of the best thermoelectric materials known so far. For comparison, this number for (Bi, Sb)_2_Te_3_ p-type TE material is 18. It is notable that according to the group theory considerations, the maximum achievable N_v_ for extrema points in a band structure is 48 which can happen in cubic crystals^43^.

**Figure 2:**
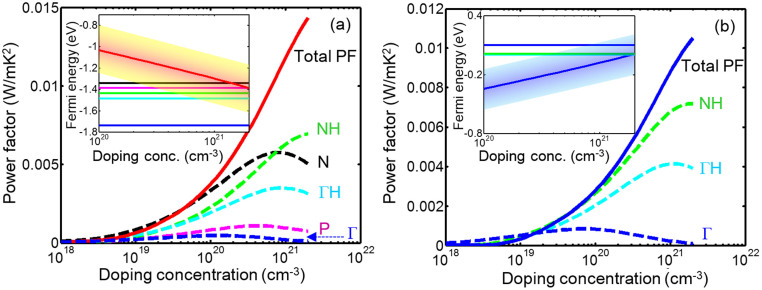
The total and the partial power factors of each peak in valence band of the type-VIII Si_46_ along with the Fermi energy as a function of doping concentration (inset). The colored band indicated the thermal broadening of the carrier distribution around Fermi energy (i.e. E_f_ ± 2k_B_T). (a) p-type, and (b) n-type.

We calculated the Fermi energy and the power factor as functions of the doping concentration for the temperature of 1000 C as shown in Figure 2. The results predict that both for p-type and n-type Si_46_–VIII there exist optimum values for the doping concentration which are approximately 1.1 × 10^21^ cm^−3^ and 1.04 × 10^21^ cm^−3^, respectively. We have considered a doping concentration of 1.1 × 10^21^ cm^−3^ for both p-type and n-type Si_46_-VIII in all other calculations. As presented in Figure 2, for both types the total power factor is predicted to increase rapidly with doping concentration. The contribution of each peak of the valence band in the power factor is also presented in Figure 2. For the p-type material the highest contribution comes from the NH and N peaks and after that the ΓH, P, and Γ peaks give their contributions at higher doping concentrations, respectively. For the n-type material, NH, ΓH, and Γ valleys have highest contribution, respectively.

Figure 2-insets show the Fermi energy with thermal spreading of 2k_B_T versus doping concentration in comparison with the position of the valence band peaks. It can be seen that, at high doping concentration (>7 × 10^20^ cm^−3^), all the valence band peaks are within 2k_B_T of the Fermi energy and can contribute to the power factor. The energy separation of the valence band peaks with respect to the N point, as a reference, are approximately 0.0449, 0.094, 0.1443, and 0.3952 eV, respectively, for the P point, points along NH and ΓH lines, and the Γ point. The reduction of the N point power factor at high doping concentration shows that the optimum doping value for this valley is around 1.8 × 10^20^ cm^−3^. Nevertheless, the trend shows that overall power factor keeps increasing with the doping concentration up to 10^21^ cm^−3^. In case of the n type material, the energy separation with respect to ΓH are 0.0125, and 0.0992 eV for the NH and Γ points, respectively.

Doping concentration in the range of 10^21^ cm^−3^ is often too large for many known semiconductors. Therefore, one may wonder if reaching this doping level is possible in Si_46_-VIII. It is known that diamond silicon can be doped up to approximately 10^21^ cm^−3^ at high temperature. Doping of the clathrates can generally be done at higher levels compared with the doping of the Si diamond phase. Type-VIII clathrate systems can be intercalated up to 8 guest alkali or alkali-earth atoms and change the structure from C_46_ to A_8_C_46_. Therefore, up to 16 electrons per unit cell can be added to the pristine Si clathrate (C_46_), which is equivalent to 1.5 × 10^22^ cm^−3^. This type of doping is called intercalation or insertion. Replacing Si atoms in the framework by group-III elements can also change the carrier concentration; hence, the structure changes from A_8_C_46_ to A_8_B_x_C_46−x_. This type of doping is called doping by substitution^44^.

In particular, for the case of Si_46_, the material has already degenerate p-type characteristics with hole concentration of ~10^21^ cm^−3^ without any extrinsic doping. This can be seen from the inset of Figure 2-a. The Fermi energy at 10^21^ cm^−3^ is −1.25 eV below the conduction band edge, which is 0.01 eV above the valance band edge. The intrinsic Fermi level is also at the valance band edge as shown in Figure 1-c (here zero is the Fermi energy). Therefore, Si_46_ without any external doping has already optimum hole concentration to make a good p-type TE material.

**Figure 3:**
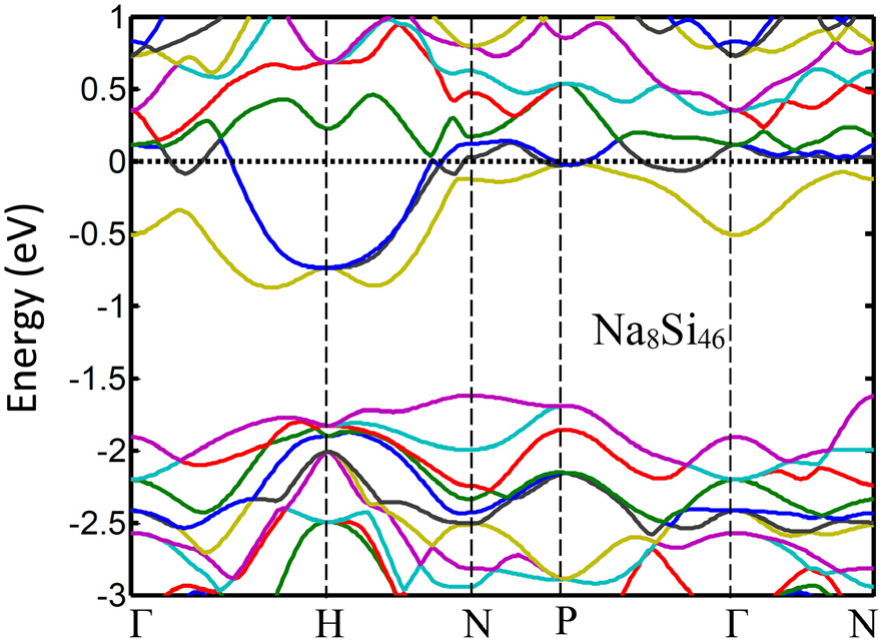
The bandstructure and position of the Fermi level (dashed line) in Na_8_Si_46_-VIII.

For n-type doping, one can use intercalation with any of the alkali or alkali-earth elements. Such intercalations can dope Si_46_-VIII to highly degenerate levels. According to Figure 2-b, at 10^21^ cm^−3^ electron concentration, the n-type Fermi level is ~−0.06 eV below the conduction band edge. The intercalation can provide large doping concentration much larger than achievable doping levels in diamond silicon which is limited to solid solubility limit of the dopants. For example, our calculations show that the Fermi level of Na_8_Si_46_-VIII is about 0.8 eV above the conduction band edge as shown in Figure 3. This is obviously much higher than the needed Fermi energy. Therefore, partial intercalation with Na can provide the required Fermi level. In addition, as it is evident from comparison of Figure 3 and Figure 1-(c), the guest atom does not affect the band structure considerably due to its weak interaction with the cage atoms. It is known that intercalation can also reduce the thermal conductivity, which is desired for TE application. Therefore, Si_46_-VIII is a good parent material for designing efficient thermoelectric materials. It is also noted that intercalation has shifted the Fermi level from the valance band edge to deep inside the conduction band. Therefore, it is possible to adjust the Fermi level anywhere in the energy gap by partial intercalation of the pristine material.

**Figure 4:**
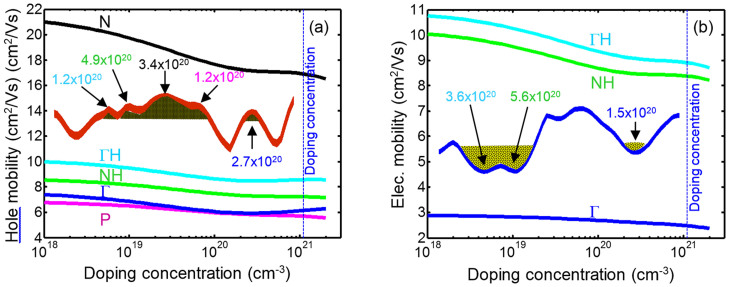
(a) The hole Hall mobility for N, ΓH, NH, Γ and P points, respectively, from high to low values and (b) the electron Hall mobility versus temperature for ΓH, NH, and Γ points, respectively, from high to low values for crystalline type-VIII Si_46_ clathrate. The vertical dashed lines indicate the level of optimum doping concentration. The inset in panel (a) shows the calculated values of hole concentration of clathrate Si_46_-VIII at each peak in the valence band. The inset in panel (b) depicts the predicted values of electron concentration of clathrate Si_46_-VIII at each valley of conduction band.

Figure 4 demonstrates the predicted charge carrier Hall mobility versus doping concentration for both p-type and n-type Si_46_–VIII clathrates in crystalline form.

Figure 4-(a) demonstrates that the hole mobility in the crystalline material decreases with the doping concentration, as a result of an increase in ionized impurity scattering. The slope of the mobility decrement versus doping concentration is almost the same for all carrier pockets while that of the N-point seems a little faster. The N-point extrema shows the largest mobility in the valence band while the P-point shows the smallest value.

Figure 4-(b) shows predictions that the valley at the ΓH-point in the conduction band possesses the highest Hall mobility and the valley at the Γ-point presents the smallest value. The slopes of Hall mobility versus doping concentration curves for ΓH and NH valleys are similar while that of Γ-point has a slower decrease. It is notable that the increment of the doping concentration leads to the contribution of more number of band extrema in the transport process and larger density of states. The larger density of states results in higher Seebeck coefficient; hence, higher thermoelectric power factor.

## Discussion

Most good thermoelectric materials are highly doped to the degenerate level. Electrical conductivity is inversely proportional to the conductivity effective mass, i.e. *m_c_ = 3/Σ_i_(1/m_i_)* in which *m_i_*'s are the principal effective masses. The Seebeck coefficient increases with the density-of-states effective mass, i.e. *m_D_ = N_v_^2/3^m_d_*, where *m_d_ = (m_1_m_2_m_3_)^1/3^* and *N_v_* is the number of equivalent valleys^45^. For a given *m_D_*, *m_c_* is smaller if the effective mass is anisotropic. Therefore, the thermoelectric power factor increases with *N_v_* and, in degenerate doped materials, with anisotropic characteristics of the effective mass^46^,^47^. For the case of Si_46_-VIII, the N-point has the highest mass anisotropy which in turn improves the power factor in this valley. Nevertheless, the N-point power factor reduces at high doping concentration due to the reduction of the Seebeck coefficient as shown in Figure 2-a.

The low lattice thermal conductivity in the clathrate compounds is due to their open framework and/or the rattling of the guest atoms in intercalated clathrates. Obviously the guest atom modes do not apply to pristine clathrates although low thermal conductivity has been also reported for other pristine Si clathrates such as Si_136_-II^35^. Therefore, the thermal conductivity of Si_46_-VIII deserves future studies. It is also expected that the thermal conductivity of the crystalline Si_46_-VIII should be reduced by the introduction of guest atoms. The guest atoms usually has small effect on electrical properties due to their weak interaction with the host atoms. Hence, a high ZT may be achieved for both n and p type structures by introducing guest atoms in the clathrate framework. For example, intercalation of clathrates by Ba and Na has been used in many clathrates in the past and can be explored for this purpose^48^,^49^,^50^,^51^,^52^.

## Conclusion

In summary, we presented the electrical transport properties of the bulk crystalline Si_46_-VIII clathrate as functions of doping concentration and temperature. The calculations were based on a combined first principles and multiband semi-classical calculations. The predicted power factor of the bulk material for p-type and n-type Si_46_-VIII clathrates were in the order of 0.014 and 0.0105 W/mK^2^, respectively, at 1000 C, which are the highest thermoelectric power factors among the existing good thermoelectric materials. The extra-large power factor of p-type and n-type Si_46_–VIII in addition to their inherent small thermal conductivity is expected to result in remarkably large ZT. While the research on clathrate systems is generally focused on their low thermal conductivity, we introduced a hypothetical clathrate material which shows the highest thermoelectric power factor compared with the available thermoelectric materials. The discovery of such a large power factor in Si_46_-VIII (and its intercalated derivatives) along with its potentially low thermal conductivity offers a direction for synthesizing efficient thermoelectric clathrate material systems.

## Supplementary Material

Supplementary Information Supplementary Info
