# Supplementary material for: Prediction of Giant Thermoelectric Power Factor in Type-VIII Clathrate Si46
Source: Sci Rep. 2014 Nov 13;4:7028. doi: 10.1038/srep07028 (PMC5382702; doi:10.1038/srep07028)
Supplement: Supplementary Information — Supplementary Info [file srep07028-s1.pdf]

Supplementary Information

Prediction of Giant Thermoelectric Power Factor in Type-VIII Clathrate Si_{46}

Payam Norouzzadeh[1], Charles W. Myles[2], and Daryoosh Vashaee[3*]

[1]Helmerich Advanced Technology Research Center, Oklahoma State University, Tulsa, OK 74106, USA
[2]Department of Physics, Texas Tech University, Lubbock, Texas 79409-1051, USA
Department of Electrical and Computer Engineering, North Carolina State University, Raleigh, NC 27606, USA
*Corresponding Author: Daryoosh Vashaee Email: daryoosh.vashaee@ncsu.edu

Contents

Supplementary Table

S1: Clathrate Si_{46}-VIII physical parameters used in the model.

Supplementary Figures

S1: Electrical conductivity and (b) Seebeck coefficient versus temperature for crystalline Si_{46}-VIII.

S2: Power factor versus temperature for both n-type and p-type crystalline Si_{46}-VIII.

Supplementary Table S1:

Table S1: Clathrate Si_{46}-VIII physical parameters used in the model[3].

Parameter	Value
Debye temperature (K)	549
Static dielectric constant	12.14
High frequency dielectric constant	12.05
Sound velocity (m/s)	5196
Lattice constant (Å)	10.10
Mass density (kg/m^3)	2.082×10^3
Energy gap (eV)	1.24
Conduction band (CB) effective mass- ΓH line	(0.89 0.89 0.49)
Conduction band (CB) effective mass- NH line	(0.7 0.7 0.55)
Conduction band (CB) effective mass- Γ point	(0.315 0.315 0.315)
Valence band (VB) effective mass- ΓH line	(0.75 0.56 0.56)
Valence band (VB) effective mass- NH line	(1.58 0.52 0.52)
Valence band (VB) effective mass- N point	(2.29 0.29 0.29)
Valence band (VB) effective mass- P point	(0.76 0.76 0.76)
Valence band (VB) effective mass- Γ point	(0.45 0.45 0.45)
CB acoustic phonon deformation potential (eV)	10
VB acoustic phonon deformation potential (eV)	10
Strain parameter for point defect scattering	40

Note 1: The three numbers in parentheses are corresponding to principal effective masses (m_1, m_2, and m_3)$\times m_e$ in which m_e is the effective mass of the free electron.
Note 2: The data in the last three rows are assumptions based on typical values for silicon.

We employed density functional theory to calculate the effective masses and dielectric constants. To obtain the effective masses, we fitted the energy bands to a quadratic polynomial. We used a dense 8×8×8 mesh around each selected k-point to increase the accuracy of the effective mass calculation from the band curvature. The dielectric permittivity is related to the second derivative of the energy with respect to an electric field. In order to calculate the static and high frequency dielectric constants for type-VIII clathrate Si_{46}, we used a linear response technique through density functional perturbation theory as implemented in VASP code.[1,2]

The input parameters used in the model can be categorized into two sets. The entire first set of the main parameters such as band structure and lattice properties parameters (listed above the dashed line in Table S1) was derived from density functional theory calculations of electronic structure, elastic, and vibrational properties of clathrate Si_{46}-VIII.[3] The effective masses were obtained by fitting the energy bands to a quadratic polynomial. The energy gap was calculated by DFT-GGA method as reported in Ref. 3. Usually hybrid functionals such as HSE06 and PBEsol give better results than LDA and GGA for the band gap calculation.[4] We applied PBEsol functional with the same setup as for GGA and found that the indirect band gap is 1.255eV which is not very different from the GGA result (1.24eV). The non-parabolicity parameters were calculated by polynomial fitting of the bandstructure. We ignored the variation of the energy gap versus temperature. The model accounted for the multiple valleys and peaks in the conduction and valance bands,

respectively, in order to determine the charge transport properties over a wide range of doping concentration and temperature.

In order to calculate the thermoelectric properties, we first solved the condition of charge conservation to find the Fermi level for a given carrier concentration and temperature. Then we calculated the relaxation times for intra-valley acoustic phonon, intervalley phonon, and ionized impurity scatterings which are the dominant scattering mechanisms. The acoustic phonon relaxation time was calculated using effective deformation potential approach.[5] The computational procedure can be found in ref. [6]. We used Thomas-Fermi approximation based on the Debye screening length and given by the Brooks-Herring formalism[7] to calculate the ionized impurity relaxation time. The standard equations are given in the literature.[7,8,9] The calculated relaxation times were combined using the Matthiessen's rule to calculate the thermoelectric properties.

Supplementary Figure

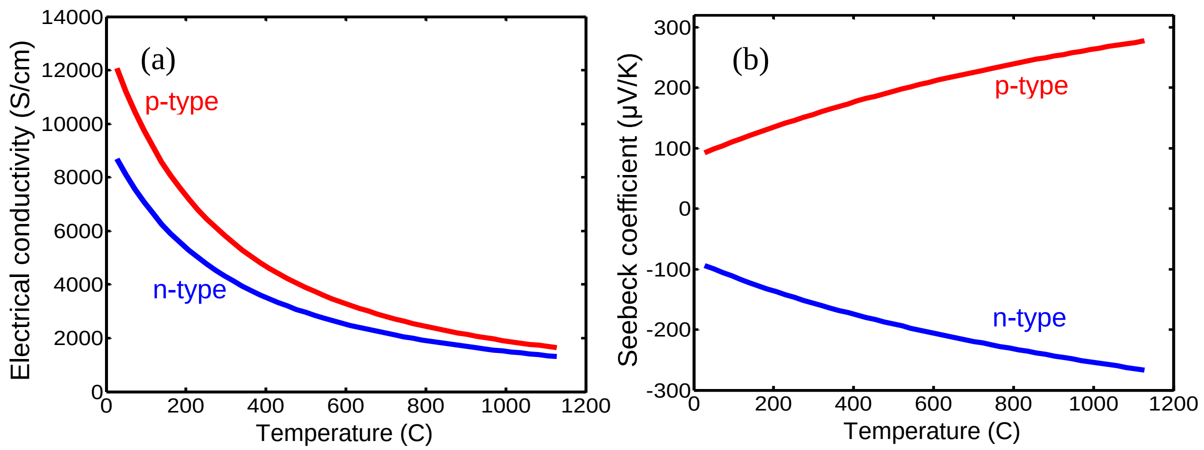

Figure S1: (a) Electrical conductivity and (b) Seebeck coefficient versus temperature for crystalline clathrate Si_{46}-VIII for both p-type (red line) and n-type (blue line) materials at doping concentration of 1.1×10^{21} cm^{-3}.

Error! Reference source not found. compares the electrical conductivity and Seebeck coefficient for both p-type and n-type Si_{46}-VIII. The smaller the electrical conductivity of the n-type Si_{46}-VIII is mainly due to its larger conductivity effective mass compared with that of the p-type material. As it can be seen in **Error! Reference source not found.**-(a), the electrical conductivity decreases smoothly with temperature and never change behavior at higher temperatures. **Error! Reference source not found.**-(b) shows the Seebeck coefficient variation as a function of the temperature for crystalline Si_{46}-VIII. Consistent with the behavior of electrical conductivity, the Seebeck coefficient increases continuously with temperature. The unchanged slope of the electrical conductivity and the Seebeck coefficient curve at higher temperature region can be attributed to the relatively large band gap of Si_{46}-VIII (i.e. $E_g>10k_BT$), which prevents the bipolar effect.

Supplementary Figure

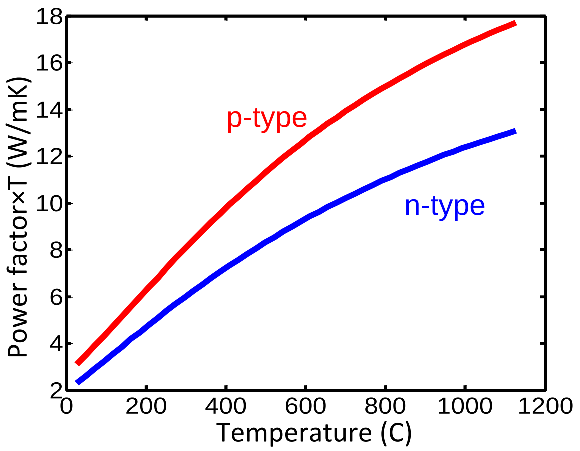

Figure S2: Power factor versus temperature for both n-type and p-type crystalline Si_{46}-VIII.

As it is illustrated in Figure S2, the thermoelectric power factor increases with temperature for both n-type and p-type Si_{46}-VIII. We chose 1000 C for our calculations.

[1] Gonze, X. et al. ABINIT: First-principles approach to material and nanosystem properties, *Computer Phys. Commun*. 180, 2582-2615 (2009).

[2] Gonze, X. et al. A brief introduction to the ABINIT software package, Kristallogr. 220, 558-562 (2005).

3. Norouzzadeh, P., W. Myles, C. and Vashaee, D. Prediction of a large number of electron pockets near the band edges in type-VIII clathrate Si_{46} and its physical properties from first principles. *J. Phys.: Condens. Matter* **25**, 475502 (2013).

4. Xiao, H., Tahir-Kheli, J., and A. Goddard, W. Accurate Band Gaps for Semiconductors from Density Functional Theory. *Phys. Chem. Lett*. **2**, 212-21 (2011).

[5] Zayachuk, D. M. The dominant mechanisms of charge-carrier scattering in lead telluride, *Semiconductors*, 31, 2 (1997).

[6] Zamanipour, Z., Shi, X., Dehkordi, A. M., Krasinski, J. S., and Vashaee, D. The effect of synthesis parameters on transport properties of nanostructured bulk thermoelectric p-type silicon germanium alloy, *Phys. Status   Solidi A*. **209**, 10 2049-2958 (2012).

[7] Lundstrom, M. Fundamentals of Carrier Transport, 2nd ed., (Cambridge University Press, Cambridge, 2000).

[8] Singh, J. Physics of Semiconductors and their Heterostructures, (McGraw-Hill, Singapore, 1996).

[9] Nag, B. R. Electron Transport in Compound Semiconductors, Springer Series in Solid-State Sciences Vol. 11, (Springer, New York, 1980).
